# Trace Element Concentrations in Follicular Fluid and Serum and Their Association with Assisted Reproductive Technology Outcomes: Preliminary Evidence

**DOI:** 10.3390/toxics14100891

**Published:** 2026-10-06

**Authors:** Giovanni Forte, Beatrice Bocca, Davide Tutedde, Patrizia Monti, Angela Sabalic, Andrea Pisano, Cristiano Farace, Claudia Trignano, Matteo Scarafuggi, Marco Petrillo, Giampiero Capobianco, Roberto Madeddu

**Affiliations:** 1 Department of Environment and Health, Italian National Institute of Health, 00161 Rome, Italy; giovanni.forte@iss.it (G.F.); beatrice.bocca@iss.it (B.B.); 2 Department of Biomedical Sciences, University of Sassari, 07100 Sassari, Italy; angelasabalic08@gmail.com (A.S.); pisanoandrea87@gmail.com (A.P.); cristiano.farace@hotmail.it (C.F.); ctrignano@uniss.it (C.T.); m.scarafuggi@studenti.uniss.it (M.S.); rmadeddu@uniss.it (R.M.); 3 International Society for Research on Cadmium and Trace Element Toxicity (ISRCT), 07100 Sassari, Italy; 4 Gynecologic and Obstetric Clinic, Department of Medical, Surgical and Experimental Sciences, University of Sassari, 07100 Sassari, Italy; patrizia.bth.ss@gmail.com (P.M.); mpetrillo@uniss.it (M.P.); capobia@uniss.it (G.C.); 5 Oncology Department, ASST Papa Giovanni XXIII, 24127 Bergamo, Italy

**Keywords:** assisted reproductive technology, metals, serum, follicular fluid, infertility

## Abstract

The World Health Organization defines infertility as a disease of the male or female reproductive system characterized by failure to achieve pregnancy after 12 months of regular unprotected intercourse. Assisted reproductive technologies (ART) are widely used to address infertility. Given the involvement of trace elements in ovarian function and female reproduction, this study evaluated their concentrations in follicular fluid and serum from 31 women undergoing ART. Concentrations of Cd, Co, Cr, Cu, Fe, Hg, Mn, Ni, Pb, Se, V, and Zn were determined by ICP-MS and investigated in relation to ART outcomes. In follicular fluid, Cu and Zn concentrations were positively associated with the number of retrieved oocytes. Conversely, Cu, V, and Zn were negatively associated with endometrial thickness, and Cu concentrations decreased with increasing fertilization rates. In serum, Co and Fe concentrations were positively associated with endometrial thickness, whereas Co and Mn were positively associated with oocyte maturation rate. Negative associations were observed between Ni concentrations and both endometrial thickness and β-hCG positivity, while V concentrations were negatively associated with β-hCG positivity and fertilization rate. Overall, the findings suggest associations between selected trace elements and ART-related parameters and support further investigation of both local and systemic metal exposure in reproductive health research. Given the exploratory nature of the study, the limited sample size, and the multiple comparisons performed, the results should be considered preliminary and hypothesis-generating and require confirmation in larger independent cohorts.

## 1. Introduction

According to the World Health Organization (WHO), infertility is a disease of the male or female reproductive system, defined as the inability to achieve pregnancy after 12 months or more of regular unprotected sexual intercourse. Affecting nearly one in six adults worldwide, infertility represents a major public health issue with significant psychological, social, and economic consequences [1]. In Italy, it is estimated to affect approximately 15% of couples [2].

Over the past three decades, assisted reproductive technologies (ART) have become increasingly important in the treatment of infertility. Today, ART accounts for approximately 2–5% of births in developed countries, with about 4.2% of live births in Italy resulting from these techniques [3,4,5].

The causes of infertility are multifactorial and include obesity, genetic predisposition, sexually transmitted infections, delayed childbearing, and lifestyle factors [6]. In addition, growing evidence highlights the role of environmental exposures, particularly metals, as potential contributors to reproductive dysfunction through interference with key physiological processes involved in female reproduction [7,8].

Both altered metal homeostasis and the accumulation of toxic metals in female reproductive organs can negatively affect reproductive capacity. Essential trace elements such as Co, Cu, Fe, Mn, Se, and Zn are involved in oogenesis, oocyte maturation, ovulation, and overall oocyte function [9,10,11,12]. However, imbalances in their levels may be detrimental: deficiencies in Fe, Se, Mn, or Cu have been associated with impaired follicular development, increased ovarian cell apoptosis, and DNA damage in maturing oocytes [13,14,15,16,17].

Conversely, excessive concentrations of essential metals may also exert harmful effects, including oxidative stress, hormonal alterations, impaired follicular development, and increased follicular atresia. In particular, elevated Fe, Zn, and Co levels have been linked to reduced ovarian function, mitochondrial dysfunction, and impaired oocyte maturation [18,19,20,21,22,23].

Exposure to non-essential and toxic elements, including Cd, Cr, Hg, Ni, Pb, and V, has been associated with reduced female fecundity, decreased ovarian reserve, altered hormone levels, and increased reproductive disorders [24,25,26,27,28,29,30,31]. These effects are mainly mediated by oxidative stress, endocrine disruption, and altered gene expression [32,33,34].

Follicular fluid (FF) is a critical component of ovarian follicle physiology, providing the biochemical microenvironment required for oocyte growth and maturation while mediating bidirectional communication between the oocyte and surrounding somatic cells [35]. As the immediate environment of the developing oocyte and the first compartment through which it is exposed to external factors, alterations in FF composition may directly influence oocyte competence, fertilization, and early embryonic development [36].

Unlike systemic matrices such as blood or serum, FF reflects the local ovarian microenvironment and therefore allows a more accurate assessment of the oocyte’s direct exposure to metals. However, serum remains an important biomarker of systemic exposure and metal homeostasis. The combined evaluation of FF and serum may therefore provide complementary information on both local and systemic factors potentially influencing oocyte quality and ART outcomes [37].

The aim of the present study was to quantify Cd, Co, Cr, Cu, Fe, Hg, Mn, Ni, Pb, Se, V, and Zn concentrations in follicular fluid and serum from women undergoing ICSI and to explore their associations with ART-related outcomes. Additional analyses were conducted to evaluate relationships between trace element concentrations and selected demographic, clinical, and reproductive variables.

## 2. Materials and Methods

### 2.1. Subjects

All 31 participants were recruited at the Gynecologic and Obstetric Clinic of the University of Sassari. Both partners underwent a standard infertility evaluation, including medical history, physical examination, assessment of ovulation through mid-luteal serum progesterone, semen analysis, and evaluation of tubal patency by hysterosalpingography or laparoscopy. All women underwent intracytoplasmic sperm injection (ICSI). Table 1 summarizes the demographic, clinical, and ART characteristics of the study population. Primary infertility was defined as the inability to achieve pregnancy after 12 months of regular unprotected intercourse (or after 6 months in women aged ≥35 years) in the absence of previous pregnancies. Ovarian reserve was assessed using anti-Müllerian hormone (AMH), follicle-stimulating hormone (FSH), luteinizing hormone (LH), and estradiol (E2) levels. The number of retrieved oocytes was recorded at oocyte pick-up, whereas mature oocytes were identified as metaphase II (MII) oocytes. The oocyte maturation rate (MR) was calculated as the proportion of MII oocytes relative to the total number of retrieved oocytes. The fertilization rate (FR) was defined as the proportion of normally fertilized oocytes, identified by the presence of two pronuclei (2PN), relative to the total number of MII oocytes. Biochemical pregnancy was defined as a positive serum β-hCG test 14 days after embryo transfer and confirmed by subsequent positive measurements. All participants were non-smokers, reported no alcohol consumption, and followed a mixed diet. Written informed consent was obtained from all participants before study enrolment.

### 2.2. Sample Collection, Preparation and Quantification of Metals

According to the clinical protocol, controlled ovarian stimulation was initiated on day 2 or 3 of the menstrual cycle using recombinant follicle-stimulating hormone (rFSH). A gonadotropin-releasing hormone (GnRH) antagonist was introduced when follicles reached a diameter of 14 mm to prevent premature ovulation. Final oocyte maturation was induced with recombinant hCG when at least one follicle reached 17 mm. Oocyte pick-up (OPU) was performed under transvaginal ultrasound guidance 36 h after hCG administration. Retrieved oocytes were fertilized by ICSI, and embryo transfer was carried out between days 3 and 5 after OPU. No more than two embryos were transferred under ultrasound guidance.

Serum and FF samples were collected on the day of oocyte retrieval. Serum is obtained by collecting blood into a tube without anticoagulants and allowing it to clot, followed by centrifugation to separate the liquid from the cellular clot and then, stored at −20 °C until analysis. FF was obtained during OPU by transvaginal ultrasound-guided follicular aspiration. To minimize contamination, only clear follicular fluid samples without visible blood contamination were included in the study. Samples of FF were centrifuged to remove cellular components, and the supernatant was aliquoted and stored at −20 °C until analysis.

For each participant, FF was collected from the not blood contaminated leading follicle with a size of ca. 19 mm. In this case, the measured trace-element concentrations should be considered representative of the sampled follicular microenvironment rather than of the entire follicular cohort. This aspect is addressed in the limitations section.

In both cases, aliquots of the two matrices were put into a 15 mL polystyrene tube (Corning, Glendale, AZ, USA) and diluted with ultrapure deionized water (Micro Pure UV, Thermo Scientific Barnstead, Langenselbold, Germany) in the ratio 1:10 prior to the analysis. A Thermo Scientific iCAP Qc inductively coupled plasma mass spectrometer (ICP-MS) (Bremen, Germany) was used to quantify ^114^Cd, ^59^Co, ^52^Cr, ^63^Cu, ^56^Fe, ^202^Hg, ^55^Mn, ^60^Ni, ^82^Se, ^208^Pb, ^51^V and ^64^Zn. To efficiently reduce polyatomic interference in analytical masses, metals were quantified using the He-pressurized QCell in the kinetic energy discrimination (KED) mode. For both matrices, procedural blanks to assess the possible exogenous metal contamination from plastics and reagents were analyzed. The addition calibration method was used to quantify the elements, and ^103^Rh at 1 ng/mL in the analytical solutions was used as an internal standard to account for possible matrix effects and instrumental drifts, respectively. All measured element concentrations were above their respective limits of detection (LoD) and were directly included in the statistical analyses.

Information on analytical details for the determination of metals and quality control data in serum and follicular fluid are reported in Tables S1 and S2 (Supplementary Materials).

### 2.3. Statistics

As data were not normally distributed, results are presented as median (50th percentile) and 5th−95th percentiles. Associations between continuous variables (e.g., metal concentrations, age, BMI, hormone levels, endometrial thickness, fertilization rate, maturation rate, MII and number of retrieved oocytes) were evaluated using Spearman’s rank correlation coefficient (ρ). A two-sided *p*-value <0.05 was considered statistically significant.

Receiver operating characteristic (ROC) curve analyses were performed as an exploratory approach to identify metal concentration thresholds associated with reproductive outcomes. The discriminative ability of each model was assessed using the area under the curve (AUC), sensitivity, and specificity. Cut-off values were determined using the Youden Index. Given the limited sample size, the absence of internal or external validation, and the exploratory nature of the study, ROC-derived thresholds should be interpreted as hypothesis-generating and not as clinically predictive cut-offs.

Multivariable linear regression (MLR) models were constructed to investigate associations between metal concentrations and continuous ART outcomes, including fertilization rate, endometrial thickness, MII, maturation rate and number of retrieved oocytes. Both the independent and dependent variables were log-transformed prior the regression analysis. Concentrations of all measured trace elements (Cd, Co, Cr, Cu, Fe, Hg, Mn, Ni, Pb, Se, V, and Zn) were simultaneously included as independent variables to account for co-exposure effects, while age and BMI were included as a priori selected confounders because of their established association with reproductive outcomes. No missing data were present in the variables included in the regression analyses. Multicollinearity was assessed using the variance inflation factor (VIF), and all values were below 2.5, indicating no relevant collinearity. Results are reported as geometric mean ratios (GMRs), obtained by exponentiating the regression coefficients, with corresponding 95% confidence intervals (95% CI). In this log-log model, the regression coefficient indicates that a 1% increase in the independent variable results in the percentage increase or decrease in the geometric mean of the dependent variable. Considering the limited observations-to-predictors ratio, the multivariable models were not intended to develop predictive models but rather to explore the direction and magnitude of the observed associations, rather than provide definitive evidence of statistical significance.

To account for multiple testing, *p*-values were adjusted using the Benjamini–Hochberg false discovery rate (FDR) procedure. Given the exploratory nature of the analyses and the relatively small sample size, both nominal and FDR-adjusted *p*-values are reported. For a given outcome, the *p*-values obtained across all trace elements within the same statistical test constituted the correction family. Thus, *p*-values derived from different statistical tests were adjusted separately as distinct families. Associations with nominal *p* < 0.05 that did not remain significant after FDR correction are presented as exploratory findings, whereas associations with FDR-adjusted *p* < 0.05 are considered statistically significant after correction for multiple comparisons.

Additional exploratory information on differences in metal concentrations across categorical variables were assessed using the Mann–Whitney U test or Kruskal–Wallis test, as appropriate, using dichotomized outcomes based on literature-derived thresholds or cohort-specific medians. A two-sided *p*-value <0.05 was considered statistically significant. In this context, fertilization rate (FR) was categorized using the ESHRE competence threshold of 65%, which closely corresponded to the cohort median (66.7%) [38]. Endometrial thickness was dichotomized at 9 mm based on previous studies identifying 8–10 mm as the minimum range associated with favorable reproductive outcomes [39,40]. As no universally accepted threshold exists for the optimal number of retrieved oocytes, the cohort median (9 oocytes) was used as the cut-off [41,42]. Oocyte MR was categorized as 100% versus <100% to evaluate factors associated with complete oocyte maturation, consistent with current laboratory performance indicators [38,43].

All statistical analyses were performed using IBM SPSS Statistics version 30.0.

## 3. Results

Table 2 reports element concentrations (ng/mL) as median (P50) and 5th–95th percentiles in FF and serum of women undergoing ICSI.

### 3.1. Follicular Fluid

Spearman analysis showed a positive correlation between Zn and number of retrieved oocytes (p_FDR_ = 0.004, ρ = 0.517), and a weak positive correlation between Cu and retrieved oocytes (*p* = 0.044, ρ = 0.369); this last association was not more present after the correction. Negative correlations were observed between V (p_FDR_ = 0.004, ρ = −0.501), Cu (*p* = 0.047, ρ = −0.360), and Zn (*p* = 0.011, ρ = −0.452) and endometrial thickness, even if data for Cu and Zn were not confirmed.

ROC analysis identified exploratory cut-off values associated with ICSI outcomes. For Zn and oocyte retrieval, concentrations above 287 ng/mL yielded an AUC of 0.736 (95% CI: 0.531–0.941; *p* = 0.024), with 93.8% sensitivity and 38.5% specificity, although this was not confirmed by the FDR correction. For endometrial thickness, thresholds of 0.124 ng/mL for V (AUC: 0.850; 95% CI: 0.710–0.990; p_FDR_ = 0.004) with 70.0% sensitivity and 9.5% specificity, and 312 ng/mL for Zn (AUC: 0.790; 95% CI: 0.632–0.949; p_FDR_ = 0.008) with 90.0% sensitivity and 33.3% specificity were identified.

MLR analysis confirmed that V (GMR: 0.772; 95% CI: 0.644–0.925; p_FDR_ = 0.003) and Zn (GMR: 0.555; 95% CI: 0.310–0.997; *p* = 0.040 but not confirmed by the correction) were negatively associated with endometrial thickness, corresponding to reductions of 23% and 45%, respectively. In contrast, Zn was positively associated with number of retrieved oocytes (GMR: 5.32; 95% CI: 1.16–27.4; *p* = 0.048), indicating a positive association, although the wide confidence interval suggests considerable statistical uncertainty; in fact, it disappeared when FDR corrected.

In addition, Cr levels were highest in the unexplained infertility group (P50: 0.13 ng/mL; *p* = 0.011), followed by male factor (P50: 0.11 ng/mL) and female factor infertility (P50: 0.07 ng/mL); these association were not confirmed when FDR corrected.

**Figure 1:**
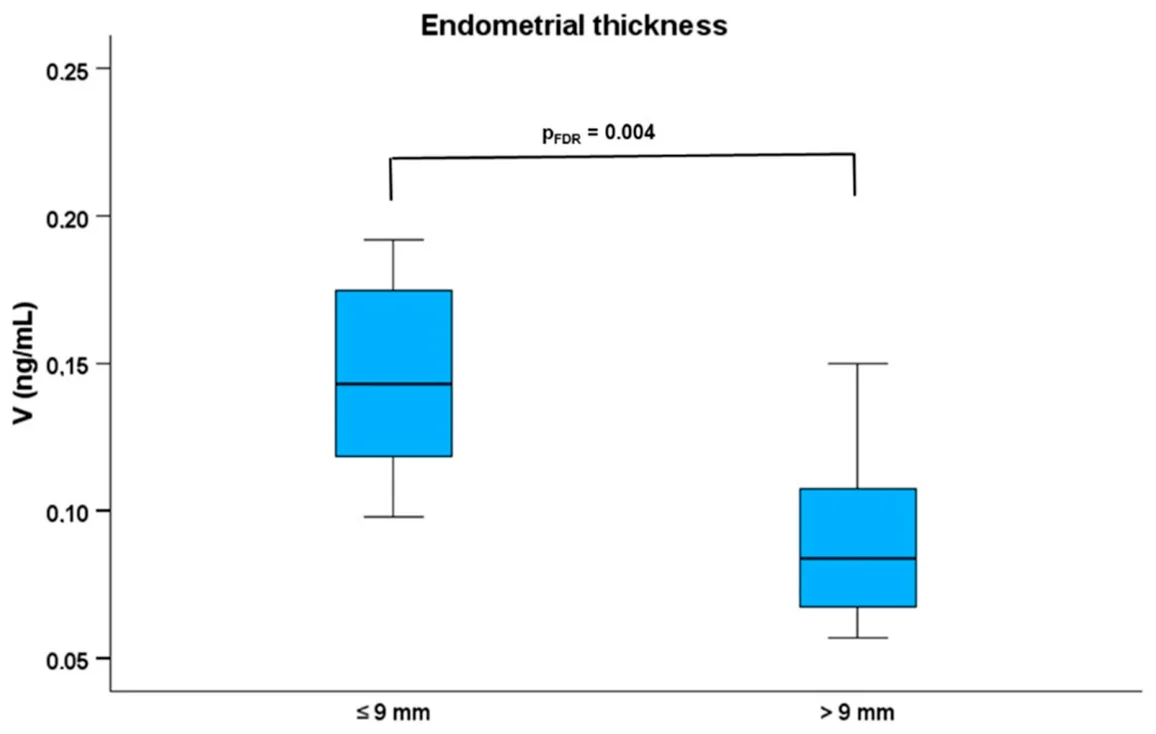
Relationship between V and endometrial thickness.

Higher FR (>65%) were associated with lower Cu (P50: 698 vs. 925 ng/mL; *p* = 0.034). Regarding endometrial thickness, Cu (P50: 695 vs. 883 ng/mL; *p* = 0.043), Mn (P50: 0.11 vs. 0.13 ng/mL; *p* = 0.029), Zn (P50: 304 vs. 326 ng/mL; p_FDR_ = 0.008), and V (P50: 0.095 vs. 0.14 ng/mL; p_FDR_ = 0.004; Figure 1) were significantly lower in women with thickness >9 mm. Associations between Cu and FR as well as endometrial thickness and Cu and Mn disappeared when the FDR was applied.

**Figure 2:**
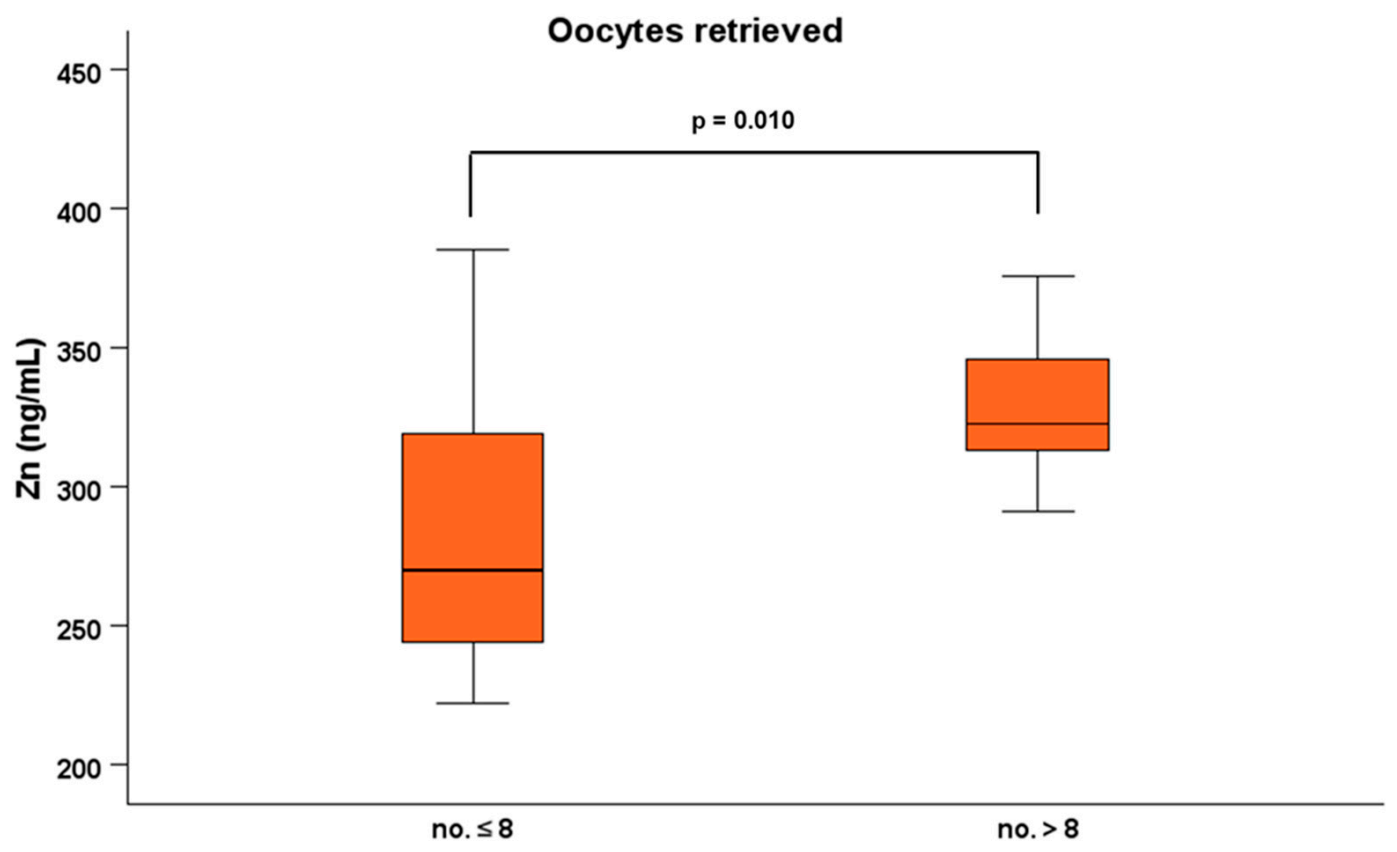
Relationship between Zn and oocytes retrieved.

Zinc was higher in women with a greater number of retrieved oocytes (P50: 322 vs. 270 ng/mL; *p* = 0.010; Figure 2). This significance did not remain after the FDR correction.

### 3.2. Serum

ROC analysis identified serum thresholds for Ni and V associated with biochemical pregnancy. Specifically, Ni concentrations below 0.34 ng/mL (AUC = 0.750; 95% CI: 0.511–0.989; *p* = 0.040) showed 100% sensitivity and 50% specificity, while V concentrations below 0.18 ng/mL (AUC = 0.799; 95% CI: 0.567–0.932; *p* = 0.012) showed 94.7% sensitivity and 25% specificity, but the FDR test did not confirm these positively.

The MLR showed significant associations between serum metal concentrations and endometrial thickness. Higher Fe (GMR: 1.30; 95% CI: 1.04–1.67; *p* = 0.027) and Co (GMR: 1.32; 95% CI: 1.02–1.71; *p* = 0.04) were associated with increases in endometrial thickness of 30% and 32%, respectively. Conversely, higher Ni was associated with a 22% decrease (GMR: 0.776; 95% CI: 0.618–0.974; *p* = 0.032). The MRL associations did not remain significant after the FDR correction.

Cadmium was the only metal that significantly discriminated among causes of primary infertility (*p* = 0.029), with higher levels in women factor (P50: 0.091 ng/mL), followed by male factor (P50: 0.046 ng/mL) and unexplained infertility (P50: 0.039 ng/mL).

Women with biochemical pregnancy showed significantly lower serum levels of V (P50: 0.18 vs. 0.22 ng/mL; *p* = 0.013) and Ni (P50: 0.35 vs. 0.48 ng/mL; *p* = 0.045) compared with the non-pregnant group.

A fertilization rate >65% was associated with lower serum V levels (P50: 0.18 vs. 0.22 ng/mL; *p* = 0.013).

Conversely, Mn (P50: 1.26 vs. 0.88 ng/mL; *p* = 0.032) and Co (P50: 0.33 vs. 0.18 ng/mL; *p* = 0.041) were significantly higher in subjects with a 100% oocyte maturation rate.

All the results obtained in the categorical analyses were not confirmed when the FDR correction was applied and, thus, considered as exploratory data.

### 3.3. Other Evidences

Strong positive correlations were observed between metal concentrations in serum and FF for Hg (ρ = 0.916; p_FDR_ = 0.002), Cu (ρ = 0.847; p_FDR_ = 0.004), Se (ρ = 0.654; p_FDR_ = 0.006), Co (ρ = 0.536; p_FDR_ = 0.008), and Fe (ρ = 0.396; *p* = 0.041). The correlation Fe–Fe did not remain significant after FDR correction.

BMI was positively correlated with duration of infertility (ρ = 0.487; p_FDR_ = 0.025), while age and AMH showed a negative correlation (ρ = −0.512; p_FDR_ = 0.017). A strong positive correlation was also observed between number of retrieved oocytes and MII oocytes (ρ = 0.856; p_FDR_ = 0.004).

Age was negatively correlated with endometrial thickness (ρ = −0.361; *p* = 0.046), consistent with multivariate regression showing a 45% reduction in thickness with increasing age (GMR: 0.55; 95% CI: 0.328–0.920; *p* = 0.026). Data were not confirmed by the FDR correction.

Finally, to check the antagonistic effect between Cu and Zn on the ART outcomes, the Cu/Zn ratio was determined in both FF and serum matrices, but results indicated that the ART parameters were not influenced when compared to the Cu/Zn ratio calculated in serum and in FF.

## 4. Discussion

### 4.1. Relation Between Metals and Art Outcomes

Follicular fluid, as the immediate microenvironment surrounding the developing oocyte, may provide valuable information on local exposure to trace elements and factors potentially involved in oocyte development and maturation. In contrast, serum measurements reflect systemic exposure and metal homeostasis. The combined evaluation of these matrices may therefore offer complementary information on local and systemic factors associated with reproductive outcomes. Follicular fluid samples were obtained from a single follicle, which may not adequately reflect intra-individual variability in trace element concentrations among different follicles within the same ovulatory cycle. As a result, caution is warranted when extrapolating these findings to the entire follicular cohort or to reproductive outcomes at the cycle level. In the present study, FF concentrations of Zn, Cu, Mn, and V, together with serum levels of Fe, Co, Ni, V, and Mn, were associated with several ICSI-related outcomes.

Zinc was significantly and positively correlated with the number of retrieved oocytes. As an essential trace element involved in DNA synthesis, cell proliferation, and enzymatic activity [44], Zn has been implicated in several processes related to folliculogenesis and oocyte competence. Previous studies have reported positive associations between FF Zn concentrations and oocyte yield, MII oocyte rates, blastocyst formation, and embryo quality [45,46]. Moreover, Zn accumulation during oocyte maturation and its release at fertilization through the “zinc spark” have been associated with oocyte activation and developmental competence [47,48]. The median FF Zn concentration observed in our cohort (313 ng/mL) was close to values previously reported in relation to embryo quality (~350 ng/mL) [49].

Conversely, this study revealed a significantly negative association between Zn concentrations and endometrial thickness. This finding contrasts with previous studies reporting positive associations between Zn status and endometrial proliferation, differentiation, and receptivity [45,50,51]. Taken together, these apparently contrasting observations suggest that the relationship between Zn and reproductive parameters may be complex.

Regarding Cu, the following observed associations between the FF metal concentrations and the ART variables were at the level of hypothesis-generating. In particular, Cu was positively associated with the number of retrieved oocytes, consistent with previous studies reporting associations between Cu concentrations and ovarian response or oocyte maturation [52,53]. Nevertheless, women with fertilization rates >65% showed lower FF Cu concentrations, indicating that the relationship between Cu and reproductive outcomes may be complex. Previous studies have reported associations between elevated Cu concentrations and oxidative stress, embryo quality, and fertilization outcomes [54,55,56,57]. Potential biological mechanisms proposed in experimental studies include apoptosis induction, cytoplasmic vacuolization, and alterations in cell membrane integrity [58]. Likewise, women with greater endometrial thickness exhibited lower FF Cu concentrations. This finding is consistent with previous reports suggesting associations between elevated Cu exposure and endometrial function, including decidualization, endometrial regeneration, and implantation processes [59,60]. Cu nevertheless remains an essential trace element involved in reproductive physiology, including angiogenesis through VEGF regulation [61]. Although the Cu/Zn ratio has been proposed as a more informative biomarker than individual metal concentrations because of the antagonistic interaction between these elements [62,63,64], no significant associations with ART outcomes were detected in the present cohort. Previous studies have reported inconsistent findings, linking elevated Cu/Zn ratios to endometrial cancer, preeclampsia, embryo quality, and biochemical pregnancy outcomes [37,57,64,65,66,67].

Among the non-essential elements, V showed the most consistent associations across both matrices. In FF, significant lower V concentrations were observed in greater endometrial thickness; while, in serum, relationships between lower V and positive β-hCG and higher fertilization rates resulted as exploratory findings.

Together, these findings indicate an inverse association between V concentrations and selected reproductive parameters. This observation is consistent with previous evidence describing V as an endocrine-disrupting metal potentially involved in alterations of steroidogenesis, ovarian follicular development, and uterine morphology [26,28,29,68]. In addition, V exposure has been associated with reduced fertility, recurrent miscarriage, and adverse pregnancy outcomes [69,70]. Although the biological mechanisms remain to be fully elucidated, the present findings support the hypothesis that V concentrations may be related to reproductive outcomes.

Serum Fe concentrations, as hypothesis-generating, were positively associated with endometrial thickness. Iron plays a crucial role in oxygen transport, cellular metabolism, and tissue vascularization [71]. Previous studies have suggested that Fe availability is related to uterine perfusion, endometrial receptivity, and endometrial growth [72]. The positive association observed in the present study is consistent with the known physiological functions of Fe in reproductive tissues.

As exploratory evidence, higher serum Co concentrations were positively associated with both oocyte maturation and endometrial thickness. Similar associations have been reported in previous studies, although the biological mechanisms underlying these observations remain poorly understood. Previous studies have reported positive associations between Co concentrations and reproductive outcomes, including higher MII oocyte rates, greater endometrial thickness, and improved markers of ovarian reserve [21,52,73]. Because Co is a constituent of vitamin B12, it is involved in several physiological processes related to cellular metabolism [71]. Nevertheless, circulating Co concentrations should not be considered a direct marker of vitamin B12 status, and the associations observed in the present study should be interpreted cautiously.

Results indicated, as hypothesis-generating, that higher serum Ni concentrations were negatively associated with a decrease in the endometrial thickness and with women having negative β-hCG outcomes. Nickel exposure has been implicated in several mechanisms potentially related to female reproductive health, including endocrine disruption, oxidative stress, ovarian dysfunction, and altered reproductive performance [30,31]. Consistent with our findings, previous studies have reported inverse associations between serum Ni concentrations and female fertility, including less favorable outcomes following assisted reproductive technologies [74,75].

Finally, Mn displayed a compartment-specific pattern. As exploratory results, this study indicated higher serum Mn concentrations in women achieving complete oocyte maturation, whereas lower FF Mn concentrations were associated with greater endometrial thickness. Mn is an essential cofactor for several enzymes involved in antioxidant defense, energy metabolism, and reproductive physiology [71,76]. Previous studies have reported associations between higher systemic Mn concentrations and ovarian response or preimplantation development [57,77], whereas elevated FF Mn levels have been associated with lower oocyte maturation outcomes [52]. Although the biological mechanisms underlying these observations remain unclear, the Mn exploratory data of the present study might support the hypothesis that systemic and follicular Mn concentrations could have distinct relationships with reproductive parameters.

### 4.2. Associations Among Physiological and Reproductive Variables

Several associations among physiological and reproductive variables were observed and were generally consistent with previous evidence.

Age was inversely associated with endometrial thickness, with younger women exhibiting greater endometrial thickness than older participants. The highest values were observed in women younger than 25 years, whereas the lowest values were found in women older than 40 years. This observation is consistent with previous reports describing a decline in endometrial thickness with advancing age, potentially related to changes in estrogen exposure and uterine vascularization [78,79].

A positive correlation was also found between BMI and duration of infertility. Although the median BMI of the study population was within the normal range, a proportion of women were overweight or obese. Previous studies have reported associations between increased BMI and reproductive disorders, including hormonal and ovulatory alterations that may contribute to longer times to conception and infertility [80,81].

As expected, AMH concentrations were negatively correlated with age. AMH is a widely recognized marker of ovarian reserve and generally declines throughout reproductive aging, reflecting the progressive reduction in the follicular pool [82,83].

Finally, a positive association was observed between the number of retrieved oocytes and the number of MII oocytes. Since MII oocytes represent the mature stage required for fertilization following ICSI, this finding is consistent with the expected relationship between ovarian response and oocyte maturity [84,85,86].

Overall, these associations were broadly consistent with previous evidence and provide contextual support for the interpretation of the exploratory associations observed between trace element concentrations and ART-related outcomes.

## 5. Strengths and Limitations

This prospective exploratory study was designed to generate hypotheses regarding the relationship between trace element exposure and ART outcomes. A major strength of the study is the comprehensive evaluation of multiple stages of the reproductive process, including ovarian response, oocyte maturation, fertilization, embryo development, and biochemical pregnancy. These intermediate outcomes cannot be directly assessed in women conceiving naturally and may provide additional information on factors associated with ART success. Another strength is the simultaneous assessment of trace element concentrations in both serum and FF. While serum reflects systemic exposure and facilitates sample collection and handling, FF represents the immediate microenvironment surrounding the developing oocyte and may provide complementary information on local trace element exposure [87]. The combined evaluation of these matrices allowed a broader characterization of trace element exposure in relation to ART-related outcomes.

Several limitations should also be acknowledged. First, the relatively small sample size may have limited the statistical power to detect weaker associations and prevented subgroup analyses according to infertility etiology, treatment characteristics, or other clinical factors. In addition, a large number of comparisons were performed across multiple elements, matrices, and reproductive outcomes. Although FDR correction was applied, the possibility of type I error cannot be completely excluded. Furthermore, ROC-derived cut-offs were generated and evaluated within the same study population and were not externally validated. Therefore, they should be considered exploratory thresholds rather than clinically applicable or predictive cut-offs.

Second, the study population consisted exclusively of infertile women undergoing ART, which may limit the generalizability of the findings to women conceiving spontaneously or to the broader reproductive-age population. Additional limitations relate to exposure assessment. Follicular fluid samples were collected from a single follicle and may not fully capture intra-individual variability in trace element concentrations across follicles [88]. Consequently, the representativeness of a single follicular measurement for cycle-wide reproductive outcomes cannot be assumed.

Furthermore, although strict procedures were implemented during sample collection, processing, and storage, environmental contamination cannot be entirely excluded [89]. Finally, trace element concentrations were measured at a single time point on the day of oocyte retrieval. Therefore, for elements characterized by short biological half-lives, the measured concentrations may not accurately reflect long-term exposure, potentially resulting in exposure misclassification.

## 6. Conclusions

This exploratory study identified associations between selected trace elements measured in serum and follicular fluid and several ART-related parameters, including oocyte retrieval, oocyte maturation, fertilization rate, endometrial thickness, and biochemical pregnancy.

Because of the limited sample size, single-center design, single time-point exposure assessment, multiple statistical comparisons, and lack of external validation, the observed associations should be considered preliminary and hypothesis-generating rather than evidence of causal effects. Although correction for multiple testing was applied, the possibility of residual confounding and chance findings cannot be completely excluded.

The findings support further investigation of trace element exposure as a potentially relevant factor in reproductive health research; however, they do not establish clinical utility for trace-element profiling in fertility management.

Further studies with larger populations, longitudinal exposure measurements, external validation, and comprehensive adjustment for potential confounding factors are needed to confirm these associations and clarify their biological significance.

## Figures and Tables

**Table 1:** Characteristics of subjects and ART parameters as median and interquartile range in brackets.

Subjects (no.)	31
Age (years)	33.0 (31.0–38.5)
BMI (kg/m^2^)	22.8 (21.9–25.5)
Duration of infertility (years)	2.00 (2.00–3.00)
Primary infertility diagnosis (no.): Unexplained Male factor Female factor	15 11 5
AMH (ng/mL)	1.70 (1.10–2.33)
FSH (mIU/mL)	7.65 (5.98–10.9)
LH (mIU/mL)	4.50 (3.48–6.55)
E2 (pg/mL)	35.0 (25.4–59.0)
Duration of stimulation (days)	10.0 (9.00–11.0)
Total dose of gonadotropins (IU)	1800 (1500–2700)
E2 on day of hCG (pg/mL)	1754 (1267–2847)
Endometrial thickness (mm)	10.0 (8.55–10.4)
Oocytes retrieved (no.)	9.00 (4.25–10.0)
Metaphase II oocytes (no.)	6.50 (4.00–8.75)
Oocyte maturation rate (%)	81.5 (70.4–100)
Fertilization rate (%)	66.7 (50.0–79.2)
Embryo transferred (no.)	2.00 (1.00–2.00)
Biochemical pregnancy (no.)	9

**Table 2:** Median and percentiles (P5−P95) concentration of metals in follicular fluid (FF) and serum expressed in ng/mL.

Metals	Follicular Fluid (FF)	Serum
Cd	0.023 (0.015–0.053)	0.046 (0.023–0.099)
Co	0.06 (0.03–0.15)	0.20 (0.15–0.51)
Cr	0.13 (0.07–0.35)	0.33 (0.23–0.84)
Cu	785 (477–1233)	1219 (965–1644)
Fe	653 (394–2207)	979 (455–1708)
Hg	0.25 (0.07–0.88)	0.37 (0.15–1.40)
Mn	0.11 (0.06–0.38)	0.97 (0.73–3.91)
Ni	0.17 (0.08–0.46)	0.46 (0.28–1.50)
Pb	0.18 (0.10–0.27)	0.11 (0.07–1.20)
Se	42.8 (31.4–54.9)	90.8 (72.8–110)
V	0.10 (0.05–0.19)	0.21 (0.17–0.32)
Zn	313 (238–409)	1128 (817–1631)
Cu/Zn ratio	2.56 (1.59–3.69)	1.09 (0.73–1.82)

## Data Availability

The dataset presented in this article are not available for privacy and ethical reasons. Requests to access the datasets should be directed to the corresponding author.

## Supplementary Materials

The following supporting information can be downloaded at: https://www.mdpi.com/article/10.3390/toxics14100891/s1, Table S1: ICP-MS performance in serum analysis; Table S2. ICP-MS performance in follicular fluid analysis.
