# Supplementary material for: Trace Element Concentrations in Follicular Fluid and Serum and Their Association with Assisted Reproductive Technology Outcomes: Preliminary Evidence
Source: Toxics. 2026 Oct 6;14(10):891. doi: 10.3390/toxics14100891 (PMC13709938; doi:10.3390/toxics14100891)
Supplement: Supplementary file 1 [file toxics-14-00891-s001.zip › toxics-4529660-supplementary.pdf]

Supplementary Materials

Trace Element Concentrations in Follicular Fluid and Serum and Their Association with Assisted Reproductive Technology Outcomes: Preliminary Evidence

Giovanni Forte [1], Beatrice Bocca [1], Davide Tutedde [2,3,*], Patrizia Monti [4], Angela Sabalic [2,3,5], Andrea Pisano [2,3], Cristiano Farace [2,3] Claudia Trignano [2,3], Matteo Scarafuggi [2,3], Marco Petrillo [4], Giampiero Capobianco [4] and Roberto Madeddu [2,3]

[1] Department of Environment and Health, Italian National Institute of Health, 00161 Rome, Italy
[2] Department of Biomedical Sciences, University of Sassari, 07100 Sassari, Italy
[3] International Society for Research on Cadmium and Trace Element Toxicity (ISRCT), 07100 Sassari, Italy
[4] Gynecologic and Obstetric Clinic, Department of Medical, Surgical and Experimental Sciences, University of Sassari, 07100 Sassari, Italy
[5] Oncology Department, ASST Papa Giovanni XXIII, 24127 Bergamo, Italy
* Correspondence: d.tutedde@studenti.uniss.it

Quantification of Cd, Co, Cr, Cu, Fe, Hg, Mn, Ni, Pb, Se, V, and Zn in follicular fluid and serum was performed using an iCAP Qc inductively coupled plasma mass spectrometer (ICP-MS; Thermo Scientific, Bremen, Germany). To minimize polyatomic interferences, measurements were carried out using a helium-pressurized QCell operating in kinetic energy discrimination (KED) mode.

Element concentrations were quantified using the standard addition calibration method. Multi-point calibration curves were prepared using pooled diluted samples and single-element reference standards (CPAchem, Bogomilovo, Bulgaria). Rhodium (^{103}Rh) was added to all analytical solutions at a concentration of 1 ng/mL as an internal standard to compensate for instrumental drift and matrix effects.

For serum analysis, the certified reference materials (CRMs) Seronorm™ Trace Elements Serum Level I and Level II were analyzed to evaluate method accuracy and precision (Table S1).

Because no certified reference material is currently available for follicular fluid, analytical performance in this matrix was assessed using both Seronorm™ Trace Elements Serum Level I and metal-spiked follicular fluid samples (Table S2).

For both matrices, limits of detection (LODs) were calculated as three times the standard deviation of repeated reagent blank measurements. The following LODs were obtained: Cd, 0.001 ng/mL; Co, 0.001 ng/mL; Cr, 0.003 ng/mL; Cu, 0.015 ng/mL; Fe, 0.060 μg/mL; Mn, 0.002 ng/mL; Ni, 0.003 ng/mL; Pb, 0.001 ng/mL; Se, 0.1 ng/mL; and Zn, 0.025 μg/mL. All measured concentrations were above the corresponding LODs. Limits of quantification (LOQs) were calculated as ten times the standard deviation of repeated blank measurements.

To minimize external contamination, all plasticware was pre-cleaned using a 2% (v/v) nitric acid solution. Procedural blanks were analyzed throughout the analytical sequence to monitor potential metal contamination originating from reagents, laboratory consumables, and sample handling.

Table S1. ICP-MS performance in serum analysis.

**Elements**	**Calibration ranges (ng/mL)**	**Seronorm Trace Element Serum Level I**		**Seronorm Trace Element Serum Level II**	
		Certified value in ng/mL (Accuracy, %)	Precision (%)	Certified value in ng/mL (Accuracy, %)	Precision (%)
**Cd**	0.010–0.4	0.135* (95)	9.3	0.122* (97)	6.8
**Co**	0.010–0.4	0.99 (108)	6.4	2.70 (109)	4.9
**Cr**	0.010–0.4	1.50 (103)	5.9	3.91 (102)	5.8
**Cu**	50–1000	1128 (92)	6.1	2175 (97)	3.3
**Fe**	50–1000	1400 (90)	4.9	2070 (92)	3.5
**Hg**	0.010–0.4	1.20 (84)	7.1	2.27 (88)	6.4
**Mn**	0.010–0.4	9.4 (101)	6.8	14.2 (100)	5.0
**Ni**	0.010–0.4	5.06 (97)	6.5	9.5 (99)	4.7
**Pb**	0.010–0.4	0.75* (101)	8.0	1.29* (99)	6.1
**Se**	5–100	95 (89)	5.5	139 (90)	4.1
**V**	0.010–0.4	0.23* (115)	7.5	0.52* (105)	6.0
**Zn**	50–1000	1460 (94)	5.3	2090 (96)	4.0

*Elements reported as informative values only

Table S2. ICP-MS performance in follicular fluid analysis.

Elements	Calibration ranges (ng/mL)	Follicular fluid spiked solution		Seronorm Trace Element Serum Level I	
		Spike level in ng/mL (Recovery, %)	Precision (%)	Certified value in ng/mL (Accuracy, %)	Precision (%)
**Cd**	0.010–0.4	0.050 (93)	5.2	0.135* (92)	7.0
**Co**	0.010–0.4	0.050 (98)	5.0	0.99 (104)	4.5
**Cr**	0.010–0.4	0.050 (104)	6.4	1.50 (109)	6.0
**Cu**	50–1000	500 (99)	3.2	1128 (95)	4.0
**Fe**	50–1000	500 (102)	2.8	1400 (102)	4.2
**Hg**	0.010–0.4	0.050 (94)	6.1	1.20 (91)	5.8
**Mn**	0.010–0.4	0.050 (93)	4.9	9.4 (105)	5.3
**Ni**	0.010–0.4	0.050 (98)	5.9	5.06 (91)	4.5
**Pb**	0.010–0.4	0.050 (95)	6.1	0.75* (89)	6.4
**Se**	5–100	50.0 (104)	4.0	95 (95)	3.9
**V**	0.010–0.4	0.050 (102)	6.5	0.23* (94)	6.5
**Zn**	50–1000	500 (105)	3.5	1460 (98)	4.2

*Elements reported as informative values only
